# Fault Tolerant Mobile Sensor Node Traversal Schemes Based on Hexagonal Coverage

**DOI:** 10.1155/2014/130793

**Published:** 2014-02-06

**Authors:** Ganala Santoshi, R. J. D'Souza

**Affiliations:** Department of Mathematical and Computational Sciences, National Institute of Technology Karnataka, Surathkal, Mangalore 575025, India

## Abstract

Mobile sensor nodes (MSNs) are equipped with locomotive can move around after having been deployed. They are equipped with limited energy. A large portion of energy is drained during the traversal. In order to extend the life time of a MSN, the traveling distance must be minimized. Region of interest (ROI) is covered with multiple MSNs using coverage based pattern movement. When a group of MSNs are deployed to cover a given ROI, all the deployed MSNs should travel an approximately equal distance. Otherwise, the MSN which travels longer distance depletes more energy compared to the MSN which travels a shorter distance. In this work we show that, ROI partition plays great role in hole free coverage and makes the MSNs have optimized movement cost with fault tolerant support.

## 1. Introduction

Mobile sensor nodes are a particular class of wireless sensor nodes. They are equipped with locomotive. Some classes of MSNs are equipped with location identification devices along with locomotive. Like static wireless sensor nodes MSNs are also encapsulated with sensor unit, power supply unit, data processing unit, data storage, and data transmission units [1, 2].

In mobile sensor networks sensor node mobility plays a key role in the execution of the application. Mobile sensor networks are extremely valuable in situations where traditional deployment mechanisms fail or not suitable.

Static sensor nodes do not move once they are deployed. Predetermined positioning of static wireless sensor nodes is flexible only when the sensor field is small and human friendly. Placement of static wireless sensor nodes might not be possible in large and in situations like hazardous and disastrous fields. MSNs that can move around after having been deployed are the most suitable in such situations.

An algorithm or a mechanism which helps a single or a group of MSNs to participate in predefined coverage related operations in sensor field is defined as mobile traversal algorithm (MTA). In this work sensor field to be monitored is rectangular in shape; it is refereed as region of interest, which is geographically partitioned into polygons in tessellation fashion. MSNs are moved along the vertices or the center points of the polygons.

Physical phenomenon within the boundaries of the polygon is monitored by the MSNs which are placed at the vertices of it or at the center points of the polygons. On completion of predetermined operations at a polygon, MSNs are moved to occupy the vertices or center points of the horizontally or vertically adjacent polygon for predetermined operations. This procedure is repeated till the entire ROI is covered.

Research in the area of coverage pattern based movement using MSNs is very useful in detection of objects in hazardous sensor fields, where human intervention might be not possible.

This paper is organized as follows. In Section 2, the problem is classified.  Section 3 details the related work. The problem is formulated in Section 4. In Section 5, experimental results and observations are detailed and in Section 6 we conclude the paper.

## 2. Problem Classification

Coverage hole free placement and MSNs traversal patterns are two different branches of research. ROI partition makes the MSNs have coverage hole free sensing and an efficient traversal pattern makes the MSNs deplete less amount of energy during their traversal. An efficient MTA should focus on both the aspects.

### 2.1. Coverage Hole Free Placement

Sensing area of sensor is circular area, limited by the radius of sensing range *r*_*s*_. Coverage hole free placement depends on structure of the polygon because the MSNs are placed at the vertices of the polygon. Distance between the two vertices is characterized by the edge weight of the polygon.

### 2.2. Optimal Traversal Pattern

This problem is very similar to well-known traveling salesman problem (TSP), where the traveling salesman visits each city exactly once starting from source and reaches destination with shortest path. On completion of predetermined operation at a polygon, MSNs are moved to occupy their positions at neighboring polygon. Each polygon here can be represented as city and the total distance traveled by the MSNs to occupy their positions in neighboring polygon can be treated as edge weight.

## 3. Related Work

Khan et al. [3] proposed a mobile traversal scheme with three MSNs. In this method sensor field is divided into equilateral triangles in tessellation fashion. Side of triangle is taken as *r* and sensing range of the MSNs is $r/\sqrt{3}$. This placement is free from coverage hole and optimal in terms of sensing range and side of the triangle. Three MSNs are placed at the vertices of equilateral triangle to 1-cover the area under it. To cover the horizontally neighboring triangle the sensor that is not adjacent to the neighboring triangle is moved to the third vertex of the adjacent triangle so that it is covered. When the sensors reach one end, they move either up or down to cover the next row. Here again, the node that is not adjacent to the next triangle is moved to cover the area under the adjacent triangle. This traversal pattern fails to minimize the total distance needs to be traveled by the MSNs. In this method only 14% of the sensed area is used for coverage purpose.

To two cover a sensor field, Purohit et al. [4] proposed a MTA using four MSNs. Sensor field is divided into regular hexagons (not in tessellation fashion). Regular hexagons are further divided into rectangle and isosceles triangles. MSNs are placed and moved along the vertices of rectangle and isosceles triangles. To cover adjacent rectangle all four MSNs are moved to occupy the vertices of adjacent rectangle. This procedure is repeated to cover the area occupied by rectangles. One MSN is kept idle and the remaining three MSNs are used to cover the area under isosceles triangle. The MSNs which are moved along the vertices of rectangle travel more distance compared to the MSNs which are moved along the vertices of isosceles triangles. This makes MSNs to have nonuniform amount of residual energy and therefore this algorithm fails to balance the total distance traveled among the deployed nodes. Four MSNs which are placed at the vertices of rectangle create coverage hole in it. This algorithm uses about 33% of the sensed area for coverage purpose with partial coverage holes.

To two cover a rectangular ROI, D'Souza and Santoshi [5] divided it into trihexagonal fashion. Three MSNs with sensing range *r*_*s*_ are placed at the vertices of equilateral triangle. Another MSN with sensing range 2*r*_*s*_ is placed at the center point of the equilateral triangle. Three MSNs with sensing range *r*_*s*_ are moved along the vertices of equilateral triangles and MSN with large sensing range is moved along the center points of equilateral triangles. This MSN traversal procedure is repeated till the entire ROI is covered. This approach shows a reduction of 56% in total distance traveled by the MSNs and about 85% reduction in extra sensed area compared with [4].

To provide dynamic degree of coverage D'Souza and Santoshi [6] designed MTA with three MSNs which are equipped with variable sensing range capabilities. This algorithm increases degree of coverage whenever the situation demands. Lower and upper bounds of total distance traveled by the MSNs are calculated and compared with existing approaches. Total distance traveled by the MSNs in this approach with 1-degree coverage shows a reduction of 68% compared to [3]. Similarly with 2 degrees of coverage it shows a reduction of 55% in total distance traveled by the MSNs compared to [4].

MTA described in [3] requires minimum three MSNs and MTA in [4] requires four MSNs. Similarly MTA described in [5] requires four MSNs (three MSNs with normal sensing range and one MSN with large sensing range). MTA described in [6] requires three MSNs with variable sensing range capabilities. All the above MTAs are number specific and no MTA provides fault tolerant support. MTA without fault tolerant support is not preferred in real-time situations because MSNs may get failed after deployment.

Mobile sensor nodes are electromechanical devices, combination of many sub-components like locomotive, location devices, sensing unit, and communication unit, battery/power management. A MSN may get failed after initial deployment due to failure of any one of the components or un-expected environmental conditions. A MSN with any failed component is not suitable for further traversal, even though its remaining components are functioning well. A MSN which fails to coordinate with remaining deployed MSNs is treated as failed node [7].

The proposed MTA focuses on both the aspects namely coverage hole free sensing and optimized movement cost. In this work a rectangular ROI of length *L* and breadth *B* is divided into hexagons in tessellation fashion to overcome the coverage hole problem. MTA is designed to provide optimized movement cost along with fault tolerant support. In the proposed MTA initially three active MSNs are deployed and function up to failure of three MSNs. Necessary conditions for ROI partition are detailed, mathematically established, and experimentally verified.

## 4. Problem Formulation

Mobile traversal algorithm is similar to finding the Hamiltonian path in ROI. Single vertex or a set of vertices in ROI represented as a vertex in Hamiltonian path.

The problem is to define a triangular grid graph. Let *G* = (*V*,  *E*) be a graph, where *V* is the set of vertices and *E* is the set of edges. Vertices of the graph are represented by the linear combination *x* **p** + *y* **q** of the two vectors **p** = (0,  1) and $q=(1/2,\sqrt{3}/2)$, where *x* and *y* are integers. The vertices are identified with pairs (*x*,  *y*) of integers and the points with Cartesian coordinates $\left(r_{s}/2)((y\mathrm{mod}2)+2x),\;\;r_{s}\sqrt{3}(y\sqrt{3}/2\right)$. A triangular grid graph is formed, where the side of the equilateral triangle is $r_{s}\sqrt{3}$.

In a triangular grid graph *G*, let *P* be the number of rows and *Q* the number of vertices of two adjacent rows which are pairwise disjoint.

**Theorem 1**

If *P* is divisible by 2 and *Q* is divisible by 3, then such triangular grid graph *G* is denoted as *G*_*T*_, in which no vertex is left unconnected.

If *Q* is not divisible by 3, then *Q* can be written as *Q* = *K* × 3 + *Y*, for some values of *Q*; the value of *Y* may be either 1 or 2. Out of *Q* number of vertices, 3*K* number of vertices are used to form *K* number of disjoint equilateral triangles. Along with *K* number of disjoint equilateral triangles *Y* number of vertices will remain unconnected. Hence to form disjoint equilateral triangles *Q* must be divisible by 3.

In order to form *K* number of disjoint equilateral triangles vertices of two adjacent rows are used. If *P* is not divisible by 2, then a single row consisting of some *Z* number of vertices will remain unconnected at the topmost row. Hence to form a triangular grid graph in which no vertex left to be unconnected, *P* must be divisible by 2 and *Q* must be divisible 3.

**Figure 1:**
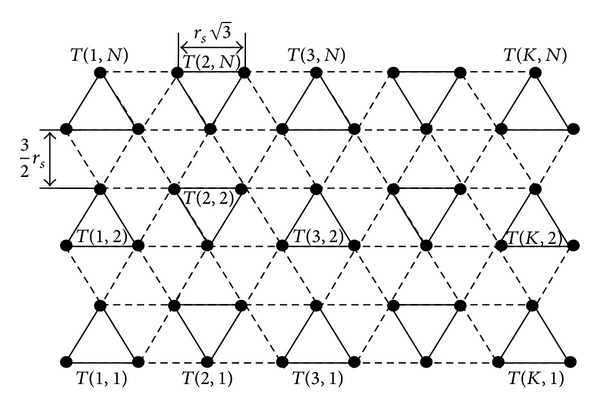
Triangular grid graph.

An equilateral triangle can be formed by connecting first two vertices of bottom row and one from its adjacent row. To make a disjoint equilateral triangle, two vertices are chosen which are horizontally adjacent to the previous triangle and connected with third vertex which is adjacent to both the vertices. Repeating this procedure *K* number of disjoint equilateral triangles are formed using the vertices of two rows in horizontal direction. This procedure is repeated till all the vertices are exhausted. The triangular grid graph will contain *N* such disjoint equilateral triangle rows. In row order, the equilateral triangles are in alternately inverted orientation and in column order, the equilateral triangles are in the same orientation, as shown in Figure 1.

**Figure 2:**
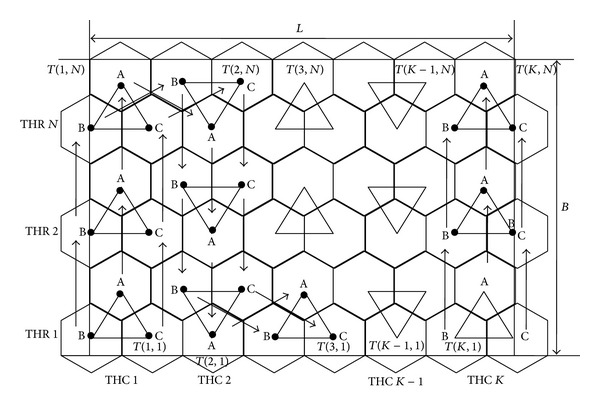
Triangle based MSNs traversal pattern.

According to Wang [8], in coverage pattern based movement, the best way to cover the sensor field is to cover it using polygons (regular hexagons), which are tiled in tessellation fashion and target locations for the mobile sensor nodes are the vertices of those hexagons. So regular hexagons of side *r*_*s*_ are drawn at every vertex in triangular grid graph as shown in Figure 2. In this work we use the word trihexagon and equilateral triangle interchangeably.

The number of equilateral triangles which are cross-sectionally adjacent to one other, in same orientation, is called trihexagonal column denoted as THC. As shown in Figure 2, all the equilateral triangles of column trihexagonal column (THC1) are same in orientation.

In the row order, the equilateral triangles are inverted alternately. The trihexagonal rows are numbered as THR 1 to THR *N* from bottom to top of the ROI. The equilateral triangles in row order are numbered from *T*(1,1) to *T*(*K*, 1). The ROI contains *N* number of trihexagonal rows and each trihexagonal row contains *K* number of equilateral triangles.

Traversal pattern with three active MSNs will be referred to as triangle based MSN traversal pattern. In triangle based MSN traversal pattern three active MSNs are traversed along the vertices of equilateral triangles. In this traversal pattern three MSNs form an equilateral triangle at every sensing point. Sensing point is a location where single or group of MSNs participate in predefined operations like coordination, sensing, and other necessary activities. The MSN movements are controlled using the method of triangulation or any suitable method supported by the GPS and location identification devices or signal from static beacons.

Source or initial sensing points in triangular pattern is *T*(1,1) and destination or final sensing point is *T*(*K*, *N*). Every trihexagonal column contains its respective first and last sensing points. Bottom sensing point of a trihexagonal column is referred to as first sensing point of that column and topmost sensing point of a trihexagonal column is referred to as last sensing point of that column.

In Figure 2, three MSN with identities A, B and C are placed at initial sensing point *T*(1,1). On completion of predetermined operations at this sensing point, they are moved to the next sensing point in that column. Similarly on completion of predetermined operations at all intermediate sensing points of that column, MSNs arrive at *T*(1, *N*). When MSNs complete the operations at the last sensing point of the column, they are moved to next column's (i.e., trihexagonal column 2) top-most sensing point that is *T*(2, *N*). Sensing procedure at all intermediate sensing points of this column is repeated as was done in previous column. Once MSNs complete the predetermined operations at *T*(2,1), they are moved to next column's (i.e., trihexagonal column 3) bottom sensing point, that is, *T*(3,1). This process is repeated, till entire ROI is sensed.

**Theorem 2**

In a triangular grid graph *G*_*T*_, the cross-sectional distance between the vertices of cross-sectionally adjacent triangles is the same as the diagonal distance between the vertices of two horizontally adjacent triangles.

In column order equilateral triangles are in the same orientation. Length of side of the equilateral triangle is $r_{s}\sqrt{3}$. Perpendicular distance between the two vertices of two cross-sectionally adjacent equilateral triangles is 3*r*_*s*_. Sum of the perpendicular distances between the three vertices of two cross-sectionally adjacent equilateral triangles is 9*r*_*s*_.

In the row order successive equilateral triangles are in inverted orientation. We define diagonal distance as the distance between a vertex of equilateral triangle and its corresponding vertex in horizontally adjacent equilateral triangle. This distance is 3*r*_*s*_. Sum of the horizontal distances between the three vertices of an equilateral triangle and their corresponding vertices in horizontally adjacent equilateral triangle is 9*r*_*s*_.

**Figure 3:**
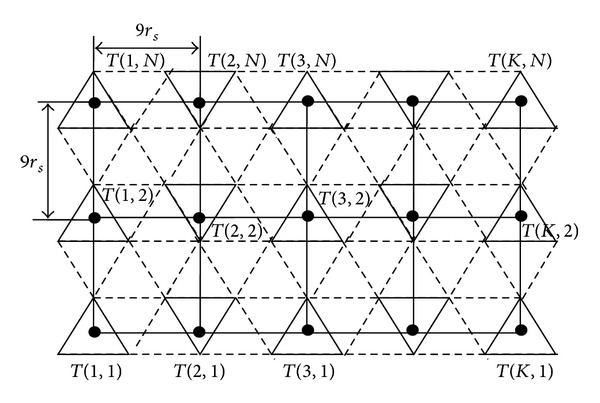
Triangular grid graph redrawn as rectangular grid graph.

Figure 1 is redrawn as Figure 3. This forms a rectangle grid graph. We define problem on rectangle grid graph *G*_*T*_ = (*V*_*T*_, *E*_*T*_), where *V*_*T*_ is set of vertices (i.e., equilateral triangles) and *E*_*T*_ is edge weight (i.e., distance between the vertices) between two adjacent equilateral triangles.

The traveling salesman problem (TSP) is defined as follows. Let *C* = (*c*_*ij*_) be the cost matrix associated with *E*_*T*_. The matrix *C* is symmetric because *c*_*ij*_ = *c*_*ji*_, for all (*i*, *j*) ∈ *C*, where *i* and *j* are two consecutive vertices (i.e., equilateral triangles). We define the following binary variable:

(1) $$\begin{array}{c} x_{ij}=\{\begin{array}{cc} 1 & \text{if}\;\text{arc}\left(i,j\right)\;\text{is}\;\text{used}\;\mathrm{on}\;\text{the}\;\text{tour},\\ 0 & \text{if}\;\text{otherwise}. \end{array} \end{array}$$

A general scheme of the assignment-based integer linear programming formulation of the TSP can be given as follows:

(2) $$\begin{array}{c} \overset{n}{\underset{i=1}{\sum }}\;\overset{n}{\underset{j=1}{\sum }}c_{ij}x_{ij}\\ \overset{n}{\underset{j=1}{\sum }}x_{ij}=1,\;\forall i\\ \overset{n}{\underset{i=1}{\sum }}x_{ij}=1,\;\forall j \end{array}$$

+ sub tour elimination constraints.

This forms a rectangular grid graph and it is connected. The (*K* × *N*) number of vertices can be connected using (*K* × *N* − 1) edges. The distance between any two consecutive vertices is 9*r*_*s*_. The distance between the source and destination remains the same whatever may be the route, since the source and destination of the traversal are fixed.

A MSN may get failed after initial deployment. A failed MSN is not suitable for further traversal. A MSN which fails to coordinate with remaining deployed MSNs is treated as failed node. A MSN may fail at any location and at any row, including the first sensing point and the last sensing point of a row in the ROI.

The coordinates of the positions of MSNs can be calculated from the satellite using GPS if the sensor field is open. If the sensor field to be monitored is closed, signals from static beacons can be used to identify the location [9].

Every traversal pattern has starting and ending sensing points. Starting point is represented as (*x*_*s*_, *y*_*s*_) and ending sensing point represented as (*x*_*f*_, *y*_*f*_). Let the starting sensing point of triangle based traversal pattern be *T*(*x*_*ts*_, *y*_*ts*_) which is also the source of the traversal, that is, *T*(1,1), and let ending sensing point of triangle based traversal pattern be *T*(*x*_*tf*_, *y*_*tf*_). If no MSN fails during the triangle based traversal, then the last sensing point of the traversal is the destination, that is, *T*(*K*, *N*). Total distance *d*_*t*_ traveled by the MSNs in triangle based pattern can be calculated as follows:

(3) dt=(xmax⁡−1)(xtf−1)ct+(ytf−1)ct+(xtf−1)ht−(xmax⁡−1)(xts−1)ct+(yts−1)ct+(xts−1)ht,

where *c* ^*t*^ is the cross-sectional distance between two triangles, *h* ^*t*^ is the horizontal distance between two triangles, and *c* ^*t*^ = *h* ^*t*^ = 9*r*_*s*_.

In a triangular grid graph *G*_*T*_ let *K* be the number of equilateral triangles in a row, and let *Q* be the number of vertices used to form *K* number of disjoint equilateral triangles.

**Theorem 3**

If *K* > 2 and is divisible by 4, then *Q*, the number of vertices used to form *K* number of disjoint equilateral triangles in triangular grid graph *G*_*T*_, can be vertex partitioned into disjoint subgraphs, each subgraph consisting of two vertices connected by an edge in which no vertex remains unconnected.

If the total number of equilateral triangles in a row is odd, then total number of vertices is also odd. The odd number of vertices is partitioned into disjoint sets of each set containing 2 vertices. This makes a single vertex remain unconnected along with some disjoint sets.

**Figure 4:**
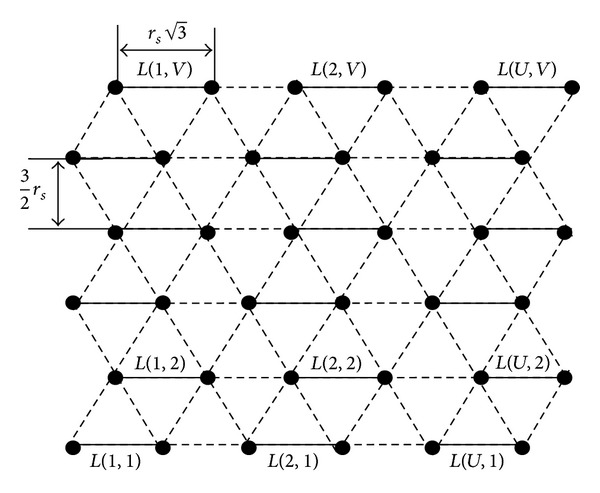
Triangular grid graph partitioned as subgraphs.

As shown in Figure 4, a graph contains *V* number of rows and each row contains *U* number of disjoint edges. In row order, disjoint edges are horizontally adjacent to each other and are numbered from 1 to *U*. *V* number of rows are numbered from 1 to *V*. Initial disjoint edge is labeled as *L*(1,1) and the final disjoint edge as *L*(*U*, *V*). Every row and column contain their respective first and last disjoint edges.

**Figure 5:**
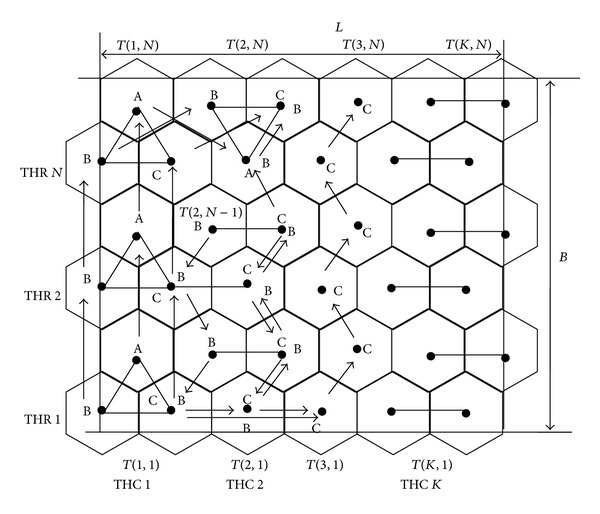
Line based MSNs traversal pattern.

Traversal pattern with two active MSNs will be referred to as line based traversal pattern. As shown in Figure 5, one MSN failed at sensing point *T*(2, *N* − 1) in triangle based traversal pattern. From this sensing point onwards the remaining two active MSNs, namely, B and C, are moved in line base traversal pattern. From this sensing point, that is, *T*(2, *N* − 1), the MSN B is moved along with MSN C, but the MSN B covers the region already being covered in triangle based traversal pattern. On successful completion of predetermined operations at all intermediate sensing points, two active MSNs reach the bottom of the ROI. From this sensing point, that is, *T*(2,1), no need to revisit any hexagons. They are moved according to line based traversal pattern, to cover the uncovered area.

We define the resulting graph as *G*_*S*_ = (*V*_*S*_, *E*_*S*_), where *V*_*S*_ is the set of vertices (i.e., subgraphs with two vertices connected with an edge) and *E*_*S*_ edge weight (i.e., distance between the vertices) between two adjacent vertices (i.e., subgraphs). Graph is shown in Figure 4.

**Theorem**

In graph *G*_*S*_, the cross-sectional distance between the two vertices is less than horizontal distance.

**Figure 6:**
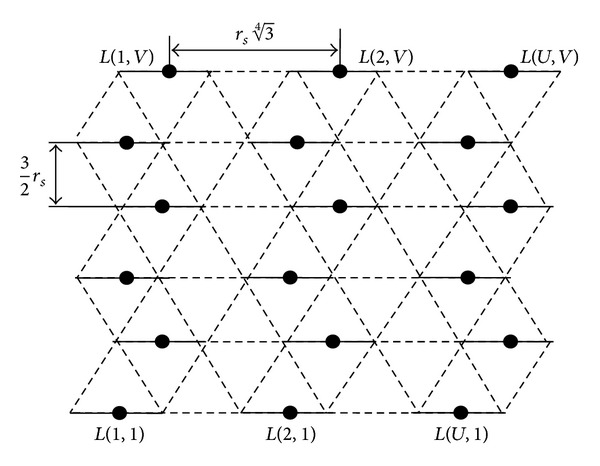
Hamiltonian path in graph *G*_*S*_.

We apply the concept of graph theory for identifying a Hamiltonian path in graph *G*_*S*_. We aim to reduce the total distance need to be traveled. The diagonal distance between the two vertices is $2r_{s}\sqrt{3}$ and the horizontal distance between two vertices is $4r_{s}\sqrt{3}$. Resultant graph is shown in Figure 6.

In the line based traversal pattern starting point is *L*(*x*_*ls*_, *y*_*ls*_) which may be an ending sensing point in triangle based traversal where a single MSN has failed. Ending point in line based traversal is *L*(*x*_*lf*_, *y*_*lf*_) which may be the starting point for vertex based traversal, that is, *V*(*x*_*s*_, *y*_*s*_). Total distance *d*_*l*_ traveled by the MSNs in line based pattern including the distance traveled by the MSNs in triangle based traversal pattern can be calculated as follows:

(4) dl=dt+(xmax⁡−1)(xlf−1)cl+(ylf−1)cl+(xlf−1)hl−(xmax⁡−1)(xls−1)cl+(yls−1)cl+(xls−1)hl,

where $c^{l}=4r_{s}\sqrt{3}$ is the cross-sectional distance between two line segments and $h^{l}=2r_{s}\sqrt{3}$ is the horizontal distance between two line segments.

**Theorem 5**

In a triangular grid graph the horizontal and diagonal distance between two adjacent vertices is the same.

Triangular grid graph is made up of equilateral triangles. Distance between any two vertices is the same. Hence the theorem is proved.

**Figure 7:**
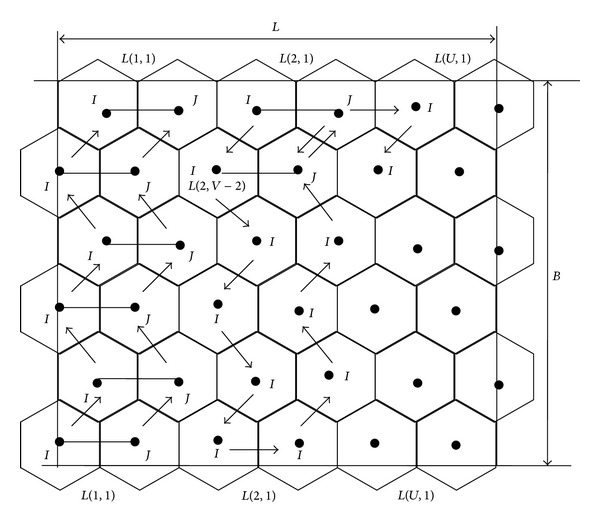
Failure of MSN in line based pattern.

As shown in Figure 7, on failure of a MSN with identity *J* at *T*(2, *V* − 1), the remaining one active MSN, namely, *I*, moves in vertex based pattern for further traversal. From this sensing point, one active MSN will move according to the predetermined path. Here the traversal pattern is changed from line based pattern to vertex based pattern, but no regular hexagons need to be revisited. Hence there are no revisits by the active MSN in this pattern. In some cases the MSN which is on right vertex of the line based traversal pattern may get failed. In such situations the active MSN which is on right vertex of the line needs to move to the left side. This makes one MSN travel a distance of one unit of traversal, that is, $r_{s}\sqrt{3}$.

Let the location where a single MSN fails in line based pattern be *L*(*x*_*lf*_, *y*_*lf*_). The starting point for vertex based traversal is *V*(*x*_*vs*_, *y*_*vs*_). This is the location where vertex based traversal pattern starts. The sensing point where the single MSN fails in vertex based traversal pattern be *V*(*x*_*vf*_, *y*_*vf*_).

Total distance *d*_*v*_ traveled by the MSNs in line based pattern including the distance traveled by the MSNs in triangle and line based traversal can be calculated as follows:

(5) dv=dt+dl+(xmax⁡−1)(xvf−1)cv+(yvf−1)cv+(xvf−1)hv−(xmax⁡−1)(xvs−1)cv+(yvs−1)cv+(xvs−1)hv,

where $c^{v}=r_{s}\sqrt{3}$ is the cross-sectional distance between two vertices and $h^{v}=r_{s}\sqrt{3}$ is the horizontal distance between two vertices. Distance between any two consecutive vertices is equal.

## 5. Experimental Results and Observations

For experimental purpose, a rectangular ROI of length *L* and breadth *B* is considered, where *L* ≫ *B*, which is divided into triangular grid graph. Regular hexagon of side *r*_*s*_ is inscribed at every vertex. Vertices in grid graph represent the locations where MSNs have to perform predetermined operations as per MTA.

The ROI is covered using *Y*_max⁡_′ rows, and each row has  *X*_max⁡_′ vertices. Columns are numbered from 1 to *X*_max⁡_′, where *X*_max⁡_′ can be determined from

(6) $$\begin{array}{c} X_{\max}^{\prime }=\frac{2L+r_{s}\sqrt{3}}{2r_{s}\sqrt{3}}. \end{array}$$

The value of *Y*_max⁡_′ can be determined from

(7) $$\begin{array}{c} Y_{\max}^{\prime }=\left[\frac{2\left(B-r_{s}\right)}{3r_{s}}\right]+1. \end{array}$$

From (6) and (7) it is clear that the value of *X*_max⁡_′ and *Y*_max⁡_′ depends on the value of *r*_*s*_. The value of *X*_max⁡_′ and *Y*_max⁡_′ needs not to be integer for all values of *L* and *B*.

To provide complete coverage along the boundaries of ROI, the values of *X*_max⁡_′ and *Y*_max⁡_′ are converted into integer using integer function. This makes some regular hexagons lie within the boundary of the ROI. In order to cover the entire ROI without coverage holes 1 is added. This makes some regular hexagons to lie on the boundary or partly outside the boundary of the ROI. With this, the area inside the ROI along with the boundaries is fully covered without coverage holes. We denote *X* and *Y* as follows:

(8) $$\begin{array}{c} X=\left[X_{\max}^{\prime }\right]+1\\ Y=\left[Y_{\max}^{\prime }\right]+1. \end{array}$$

ROI is covered using *Y*_max⁡_ number of lines and each line contains *X*_max⁡_ number of vertices. According to Theorem 1, all *X*_max⁡_ number of veritices can be used to form triangular grid graph, in which ROI contains *N* number of trihexagonal rows and each trihexagonal row contains *K* number of triangles. The values of *K*, *N*, *X*_max⁡_, and *Y*_max⁡_ are related as follows:

(9) $$\begin{array}{c} K=\frac{2\times X_{\max}}{3}\\ N=\frac{Y_{\max}}{2}. \end{array}$$

In line based partition as shown in Figure 7, ROI contains *V* number of lines and each line contains *U* number of line segments. According to Theorem 3, the values of *U*, *V* and *X*_max⁡_, and *Y*_max⁡_ are related by

(10) $$\begin{array}{c} U=\frac{X_{\max}}{2}\\ V=Y_{\max}. \end{array}$$

### 5.1. MSN Traversal without Fault Tolerance

In the first phase of simulation no failure case is considered. In triangle based traversal pattern all three MSNs are used to cover the entire ROI. In line based pattern two active MSNs are used to cover the ROI and in case of vertex based traversal single MSN is deployed to cover the entire ROI.

In triangle based traversal pattern, the ROI contains *K* × *N* number of equilateral triangles. Using (3) the total distance needed to be traveled by three active MSNs to cover the entire ROI can be written as

(11) $$\begin{array}{c} d_{tf}=9r_{s}\left(K\times N-1\right). \end{array}$$

In line based traversal pattern two active MSNs are used to cover the entire ROI. In line based pattern the ROI is divided into *U* number of rows, each row containing *V* number of line segments. Using (4) the total distance traveled by the two MSNs to cover the entire can be written as

(12) $$\begin{array}{c} d_{lf}=2r_{s}\sqrt{3}U\left(V-1\right)+4r_{s}\sqrt{3}\left(U-1\right). \end{array}$$

Similarly in the case of vertex based traversal pattern, a single MSN travels along the vertices of regular hexagons to cover the entire ROI. The distance between two adjacent regular hexagons is $\sqrt{3}r_{s}$. Total distance traveled by a single MSN to cover the entire can be written as

(13) $$\begin{array}{c} d_{vf}=\sqrt{3}r_{s}\left(X_{\max}\times Y_{\max}-1\right). \end{array}$$

All simulations are carried out using network simulator (NS2) and MATLAB software. For simulation purpose rectangular ROI of measure 9000 × 4000 units is considered. Algorithms to mobilize the MSNs are detailed in Algorithms 1 and 2. In Algorithm 1, number of active MSNs and their current locations are calculated. This algorithm takes care of calculating active MSNs and coordination among them. In case of failure of a MSN, it calculates the remaining active number of MSNs and passes the necessary information to Algorithm 2. Algorithm 2, controls the traversal pattern of the MSNs and mobilizes the MSNs between the starting and ending sensing points of every traversal through intermediate sensing points.

Simulation is carried on different values of sensing range *r*_*s*_. Optimum values of sensing range *r*_*s*_, which are suitable for triangle based traversal, line based traversal, and vertex based traversal, are considered for simulating all types of traversal patterns. We denote the total distance traveled by the MSNs in triangle based pattern as *d*_*t*_, line based pattern as *d*_*l*_, and vertex based pattern as *d*_*v*_.

The values of total distance traveled by the MSNs in different traversal patterns are tabulated in Table 1. When the sensing range is 17.38 units the total distance traveled by the MSNs in triangle based pattern is 609286, in line based pattern is 356568, and in vertex based pattern is 352143. In all the cases, the value of total distance traveled by the MSNs decreases gradually with the increase of value of *r*_*s*_. When *r*_*s*_ equals 62.60 units the total distance traveled by the MSNs in triangle based pattern is 172975, in line based pattern is 104313, and in vertex based pattern is 100084.

From Table 1 it can be observed that, when the sensing range is 17.38 units, line based traversal pattern shows reduction of 41.48% in total distance traveled by the MSNs compared to triangle based traversal pattern. Line based traversal pattern shows a reduction of 39.69%, at the value of sensing range 62.60 units.

As the sensing range increases the total distance traveled by the MSNs in line based traversal shows a decrease in distance traveled compared to triangle based traversal pattern.

When the value of sensing range is 17.38 units, vertex based traversal shows a reduction of 1.24% compared to the total distance traveled by the line based traversal pattern. Vertex based traversal shows a reduction 4.05% compared to the line based traversal, at the value of sensing range 62.60 units. As the sensing range increases, vertex based traversal shows increase in percentage of total distance traveled by a single MSN compared to line based traversal.

**Figure 8:**
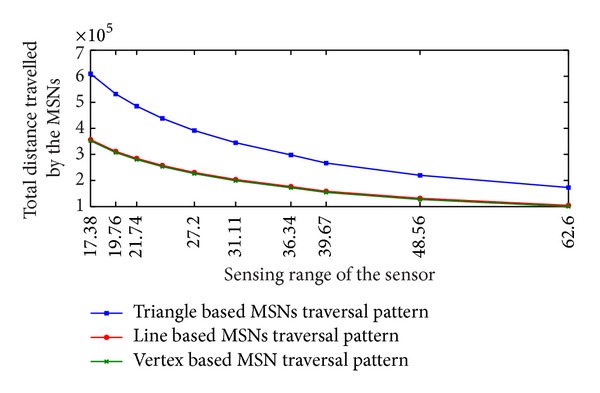
Total distance traveled by MSNs in triangle, line, and vertex based traversal.

From Table 1 and Figure 8 it can be observed that total distance traveled by the MSNs in triangle based pattern and line based pattern shows a difference of 41.48% and decreases to 39.69% with the increase of sensing range from 17.38 units to 62.60 units. It can also be observed that the distance traveled by the MSNs in line based pattern and vertex pattern is nearly equal. The difference between the line and vertex based traversal varies from 1.24% to 4.05% with the increase of sensing range from 17.38 units to 62.60 units.

The relation between *d*_*t*_, *d*_*l*_, and *d*_*v*_ can be established using (9) and (10) as follows:

(14) $$\begin{array}{c} 9r_{s}\left(X_{\max}Y_{\max}-1\right)>r_{s}\sqrt{3}X_{\max}Y_{\max}+r_{s}\sqrt{3}\left(X_{\max}-3\right)\\ 9r_{s}c-1>r_{s}\sqrt{3}c+r_{s}\sqrt{3}\left(X_{\max}-3\right), \end{array}$$

where *c* = some constant for (*X*_max⁡_ *Y*_max⁡_):

(15) $$\begin{array}{c} r_{s}\sqrt{3}X_{\max}Y_{\max}+r_{s}\sqrt{3}\left(X_{\max}-3\right)>r_{s}\sqrt{3}\left(X_{\max}Y_{\max}-1\right)\\ r_{s}\sqrt{3}c+r_{s}\sqrt{3}\left(X_{\max}-3\right)>r_{s}\sqrt{3}c-1. \end{array}$$

Equation (14) shows the difference between triangle and line based traversal. The difference between the triangle based traversal pattern and line based traversal pattern is $(9-\sqrt{3})X_{\max}Y_{\max}$. This huge difference in values of triangle based traversal and line based traversal makes the curves apart from each other. As the sensing range increases, the value of *X*_max⁡_ *Y*_max⁡_ decreases. With the increase of sensing range the lines showing the values of total distance traveled by the MSNs in triangle based traversal pattern and line based traversal pattern come closer.

From (15), it can be calculated that the difference between the line and vertex based traversal is small minimum of $r_{s}\sqrt{3}(X_{\max}-1)$. This difference remains throughout the simulation. Hence in Figure 8 lines showing line and vertex based traversal are very close to each other. The value of $r_{s}\sqrt{3}(X_{\max}-1)$ remains nearly constant for all the values of *r*_*s*_; this value is equivalent to length *L*. As the sensing range increases, the value of $r_{s}\sqrt{3}$ increases. The value of $r_{s}\sqrt{3}$ is needed to be reduced from the value of *L*.

All MSNs have equal amount of battery power at the time of deployment. In Table 2, distance traveled by the MSN in three different traversal patterns is computed and tabulated. The values shown are related to energy efficiency and calculated only in terms of distance traveled by the MSN in different traversal patterns. No concern is paid towards depletion of energy during sensing operations, communication, coordinations, and other related operations. A MSN depletes maximum amount of energy during the traversal and depletes considerable amount of energy during other operations.

When sensing range is 17.38 units, distance traveled by a single MSN in triangle based traversal is 230275 units, and in line based traversal is 178284 units, in vertex based traversal is 352143 units. At this value of sensing range, MSN in triangle based traversal shows a reduction in distance traveled by 50.63% and MSN in line based traversal shows a reduction of 57.73% in total distance traveled compared to the total distance traveled by the MSN in vertex based traversal.

Similarly at this value of sensing range, residual energy in MSN in triangle based and line based traversal is 49.38% and 42.27%, respectively. This shows that MSN which travels in triangle based traversal depletes less amount of energy during traversal and saves more energy compared to two other patterns.

When the sensing range is 62.60 units, distance traveled by the MSN in triangle, line, and vertex based traversal is 57658 units, 52156 units, and 100084 units. At this stage MSN in triangle based traversal shows a reduction of 52.11% in total distance traveled and in line based traversal it shows a reduction of 57.61% in total distance traveled compared to vertex based traversal. At this value of sensing range, residual energy in MSN in triangle and line based traversal is 47.89% and 57.61%, respectively.

On an average MSN in triangle based traversal shows 51.15% of reduction in total distance traveled and energy saving of 48.69% and in line based traversal it shows a reduction of 57.69% in total distance traveled and an energy saving of 42.19% compared to vertex based traversal.

### 5.2. MSNs Traversal with Fault Tolerance

In second phase of simulation MSNs with failure are considered. Total number of vertices in ROI is equally divided into three groups. First set of vertices are traversed using three MSNs by triangle based traversal pattern. Second group of vertices are covered using two MSNs in line based traversal pattern and remaining ROI is covered by single MSN in vertex based traversal pattern.

When the sensing range is 17.38 units, total distance traveled by the MSNs in tolerant traversal is 558391. At this value of sensing range the total distance traveled by the MSNs in triangle based traversal pattern is 609286 and in line based traversal is 356568.

When the sensing range is 62.60 units, total distance traveled by the MSNs in triangle based traversal is 172975 units. At this value of sensing range the total distance traveled by the MSNs in fault tolerant traversal is 160482.

Total number of vertices in ROI is *X*_max⁡_ × *Y*_max⁡_, which are equally distributed for three different traversals. *X*_max⁡_ *Y*_max⁡_/3 number of vertices are allotted for each type of traversal. Using (3), total distance traveled by the three MSNs in triangle based pattern with *X*_max⁡_ *Y*_max⁡_/3 vertices can be written as (1/3)(*d*_*tf*_). Similarly using (4) and (5), distance traversed by the MSNs in line based traversal and vertex based traversal with *X*_max⁡_ *Y*_max⁡_/3 vertices is (1/3)(*d*_*lf*_) and (1/3)(*d*_*vf*_). Total distance traveled by the MSNs to cover the area occupied by the *X*_max⁡_ *Y*_max⁡_ number of regular hexagons can be written as

(16) $$\begin{array}{c} d_{T}=\frac{1}{3}\left(d_{tf}+d_{lf}+d_{vf}\right)=7\sqrt{3}\left(X_{\max}Y_{\max}\right). \end{array}$$

**Figure 9:**
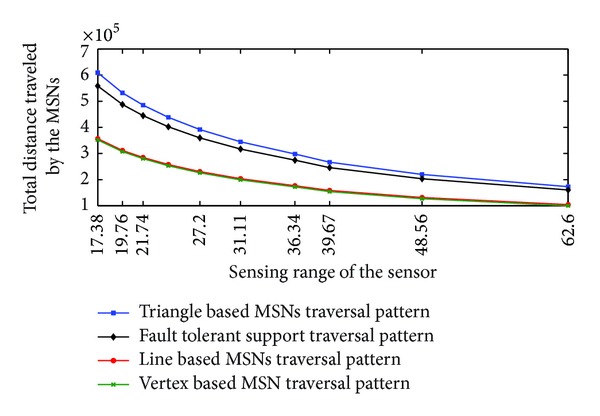
Distance traveled by the MSNs in fault and nonfault tolerant traversal.

Total distance traveled by the MSNs in fault tolerant traversal is $7\sqrt{3}(X_{\max}\times Y_{\max})$. So it lies above the values of line based traversal $r_{s}\sqrt{3}(X_{\max}Y_{\max})$ and below the values of triangle based traversal 9(*X*_max⁡_ *Y*_max⁡_ − 1). Experimental results are tabulated in Table 3 and shown in Figure 9.

Experimental results of fault tolerant and nontolerant traversal are tabulated in Table 4. When the sensing range is 17.38 units, total distance traveled by the MSNs in triangle based traversal pattern is 609286 units without fault tolerance. At this value of sensing range total distance traveled by the MSNs in fault tolerant traversal is 558391 which shows reduction of 8.35% in total distance traveled. Fault tolerant traversal shows a reduction of 7.22% compared to the total distance traveled by the MSNs in triangle based traversal at the value of sensing range 62.60 units. As the sensing range increases from 17.38 units to 62.60 units the percentage of reduction decreases from 8.35% to 7.22%.

On the other hand, when the sensing range is 17.38 units, line based traversal shows a reduction of 36.14%. At this value of sensing range the value of fault tolerant traversal is 558391 and line based traversal is 356568. When the sensing range is 62.60 units, total distance traveled in fault tolerance traversal is 160482 units and in line based traversal is 104313 units. It shows a reduction of 35.00%. As the sensing range increases from 17.38 units to 62.60 units, the percentage of reduction decreases from 36.14% to 35.00%.

The total distance traveled by the MSNs in fault tolerant system is the sum of the distance traveled by the MSNs in triangle based travel *d*_*t*_, line based travel *d*_*l*_, and vertex based travel *d*_*v*_. The value of each travel depends on the number of sensing points that are covered in that traversal pattern. In the above case, the total number of sensing points (*X*_max⁡_ *Y*_max⁡_) is uniformly distributed among all three types of traversals.

Let *h*_*t*_ be the number of sensing points covered in triangle based traversal pattern. *h*_*l*_ and *h*_*v*_ are the number of sensing points covered in line based traversal and vertex based traversal, respectively. It can be written as

(17) $$\begin{array}{c} \left(X_{\max}\times Y_{\max}\right)\leq h_{t}+h_{l}+h_{v} \end{array}$$

From (17), it can be observed that, if no MSN fails in triangle based traversal, then (*X*_max⁡_ × *Y*_max⁡_) = *h*_*t*_ and the value of *h*_*l*_ and *h*_*v*_ becomes zero. In such a case total number of sensing points covered in triangle based traversal is *h*_*t*_. If MSNs fail in triangle based traversal pattern, then the remaining sensing points will be covered in line based traversal if no further failure occurs. If MSNs fail in line based traversal, the remaining sensing points will be covered in vertex based traversal until the failure of remaining single active MSN.

The relation between the *d*_*t*_, *d*_*l*_, and *d*_*v*_ can be established as follows. The values of *d*_*t*_, *d*_*l*_, and *d*_*v*_ can be found from (3), (4), and (5):

(18) $$\begin{array}{c} 9\left(X_{\max}Y_{\max}-1\right)>7\sqrt{3}\left(X_{\max}Y_{\max}\right)>r_{s}\sqrt{3}\left(X_{\max}Y_{\max}\right). \end{array}$$

The above equation shows that vertex based traversal pattern is better and serves as lower bound and the triangle based traversal pattern serves as upper bound for fault tolerant traversal. The same lower and upper bounds are applicable for traversal pattern without fault tolerant system. Figure 9 graphically represents the lower and upper bounds of the traversal.

But from the experimental results tabulated in Table 2, it can be ascertained that the MSNs consume more energy in vertex based traversal and they serve as upper bound. Energy consumption is less in triangle based traversal pattern and it serves as lower bound.

In fault tolerant coverage based pattern movement with three MSNs, the energy depletion per MSN is less, due to traversal pattern designed for uniform energy depletion. But the total distance traveled by all the MSNs is more. When a single MSN is considered, the energy depletion is more and it travels less distance compared to other traversal patterns. In vertex based traversal or single MSN based traversal the fault tolerance is not applicable. Fault tolerance is applicable only when the deployed MSNs are more than one. In real-time systems, application with fault tolerance is highly preferred.

## 6. Conclusion

In this work ROI is divided into regular hexagons for coverage based pattern mobile sensor node movement. MSNs are moved along the centre point of the regular hexagons. Total distance traveled by the MSNs in fault tolerant and nonfault tolerant traversal is calculated. The value of total distance traveled by the MSNs in fault tolerant traversal is less compared to the total distance traveled by the MSNs in nonfault tolerant system. In the future we propose to extend our research to random deployment based mobile sensor node movement.

## 7. MSNs Traversal Algorithms

In this section we have shown two algorithms in abstract form. Total number of MSNs which are in working condition are referred to as MSN_active_ ← 3 this value is updated before the sensing operations. Each MSN calculates its current coordinate position; it can also calculate the next coordinate where it has to move.

Value of number of active MSNs is updated regularly and returned on the event of failure of MSNs as shown in Algorithm 1. In Algorithm 2 we denote *X* ^UB^ and *Y* ^UB^ as upper bounds of coordinates of the corresponding traversal. Every traversal pattern has its own upper and lower bounds for the coordinates as shown in Section 5.

Failure locations, starting points, and ending points of traversal patterns are controlled using internal algorithms with the help of signals from static beacons or GPS. Conversion of coordinates from triangle based traversal pattern to line based traversal pattern and from line based traversal pattern to vertex based traversal patterns is done implicitly.

## Figures and Tables

**Algorithm 1:**
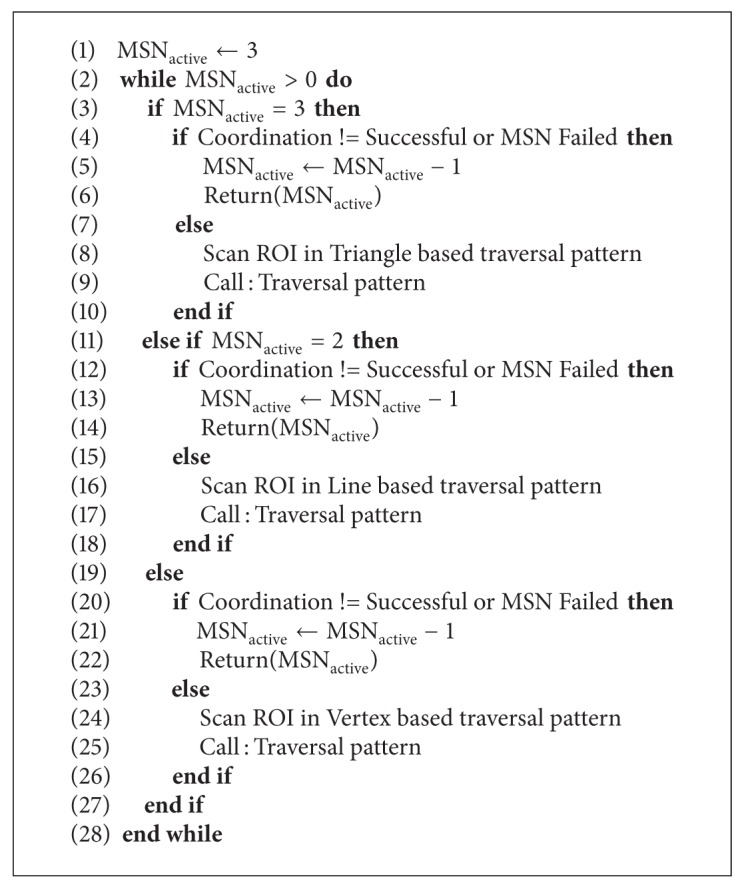
Multiple mobile traversal algorithm.

**Algorithm 2:**
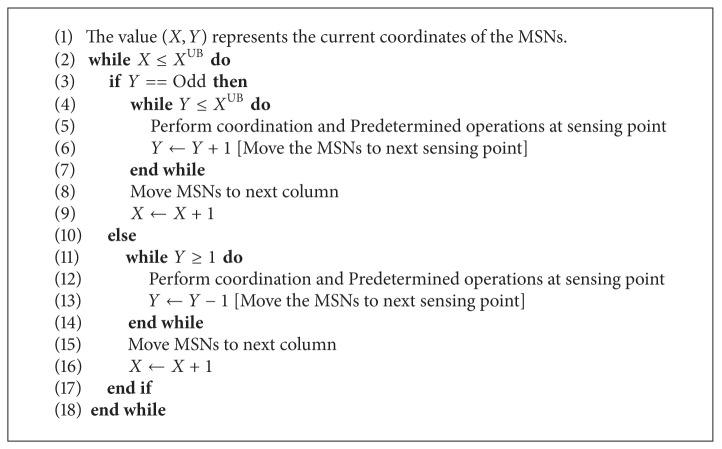
MSNs traversal algorithm—common traversal pattern.

**Table 1:** Total distance traveled by MSNs in triangle, line, and vertex based traversal.

*r*_*s*_	*d*_*t*_	*d*_*l*_	*A*	*d*_*v*_	*B*
17.38	609286	356568	41.48	352143	1.24
19.76	531844	311543	41.42	307129	1.42
21.74	485068	284535	41.34	280129	1.55
24.17	438289	257525	41.24	253130	1.71
27.20	391506	230513	41.12	226130	1.90
31.11	344719	203497	40.97	199131	2.15
36.34	297924	176475	40.77	172132	2.46
39.67	266669	158427	40.59	154099	2.73
48.56	219840	131383	40.24	127093	3.27
62.60	172975	104313	39.69	100084	4.05

*A*: % of difference between *d*_*t*_ and *d*_*l*_. *B*: % of difference between *d*_*l*_ and *d*_*v*_.

**Table 2:** Energy depletion, residual energy in different traversal patterns of MSNs.

*r*_*s*_	*d*_*v*_	*d*_*l*_	*l*_*d*_	*l*_*r*_	*d*_*t*_	*t*_*d*_	*t*_*r*_
17.38	352143	178284	50.63	49.38	230275	57.73	42.27
19.76	307129	155771	50.72	49.28	177281	57.72	42.28
21.74	280129	142267	50.79	49.21	161689	57.72	42.28
24.17	253130	128762	50.87	49.13	146096	57.72	42.28
27.20	226130	115256	51.97	49.03	130502	57.71	42.29
31.11	199131	101748	51.50	48.90	114906	57.70	42.30
36.34	172132	88237	51.26	48.74	99308	57.69	42.31
39.67	154099	79213	51.40	48.60	88889	57.68	42.32
48.56	127093	65691	51.69	48.31	73280	57.66	42.34
62.60	100084	52156	52.11	47.89	57658	57.61	42.39

*l*_*d*_, *t*_*d*_ energy depletion by the MSN in line and triangle based traversal and *l*_*r*_, *t*_*r*_ residual energy with MSN in line and triangle based traversal, respectively.

**Table 3:** Total distance traveled by the MSNs in fault tolerant traversal.

*r*_*s*_	*C*	*d*_*t*_/3	*d*_*l*_/3	*d*_*v*_/3	*D*
17.38	11700	203327	237672	117391	558391
19.76	8976	177281	207650	102422	487354
21.74	7440	161624	189640	93464	444729
24.17	6048	146168	171627	84390	402186
27.20	4800	130502	153612	75439	359554
31.11	3696	114813	135592	66502	316908
36.34	2736	99417	117566	57398	274382
39.67	2244	88889	105526	51458	245874
48.56	1512	73426	87476	42393	203295
62.60	924	57470	69397	33614	160482

*C*: total number of vertices in ROI, that is, *X*_max⁡_ × *Y*_max⁡_. *D*: total distance traveled by the MSNs with fault tolerant support.

**Table 4:** Comparison of distance traveled by the MSNs in triangle based traversal, fault tolerant traversal, and line based traversal.

*r*_*s*_	*d*_*t*_	*d*_*tf*_	*E*	*d*_*l*_	*F*
17.38	609286	558391	8.35	356568	36.14
19.76	531844	487354	8.37	311543	36.07
21.74	485068	444729	8.32	284535	36.02
24.17	438289	402186	8.24	257525	35.97
27.20	391506	359554	8.16	230513	35.89
31.11	344719	316908	8.07	203497	35.79
36.34	297924	274382	7.90	176475	35.68
39.67	266669	245874	7.80	158427	35.57
48.56	219840	203295	7.53	131383	35.37
62.60	172975	160482	7.22	104313	35.00

*E*: % of difference between *d*_*t*_ and *d*_*tf*_. *F*: % of difference between *d*_*tf*_ and *d*_*l*_.
